# Development of a wireless electroretinogram recording system

**DOI:** 10.1038/s41598-025-86750-0

**Published:** 2025-02-10

**Authors:** Tony T. C. Man, Yolanda W. Y. Yip, Chi Pui Pang, Mårten Erik Brelén

**Affiliations:** Department of Ophthalmology and Visual Sciences, The Chinese University of Hong Kong, Hong Kong Eye Hospital, 4/F, 147K Argyle Street, Kowloon, Hong Kong

**Keywords:** Biomedical engineering, Preclinical research, Translational research

## Abstract

A novel device consisting of amplifiers, an analogue-digital converter, offset correction units, and a microcontroller with Bluetooth wireless functionality was fabricated. Electroretinography (ERG) recordings were captured in dark-adapted and light-adapted full-field flash stimulations in an anaesthetised animal model using the novel wireless system. Recordings were repeated using a standard ERG recording setup of the Espion E3 system for comparison. The electroretinogram signal a-wave and b-wave amplitudes, peak times, signal offset, potential drift, and power line noise were compared between the recording setups. Signals from the novel system were found to have similar waveforms to those recorded from the standard ERG setup. There were no significant differences in the a-wave amplitudes (*P* > 0.43), a-wave peak times (*P* > 0.61) and b-wave peak times (*P* > 0.55). The offset potential drift when using the novel system was significantly smaller than the reference system (*P* < 0.02). The novel system also showed stronger resilience against powerline noise interference, as evidenced by a statistically significant performance increase during recordings with substantial noise interference (*P* < 0.01). The on-source signal processing and wireless transmission can improve the quality of recorded ERG signals. Our study demonstrates a proof of concept for performing wireless ERG recordings, which enhance the performance by reducing noise and potential drift in the recorded signals.

## Introduction

Electroretinography (ERG) is a technique for recording changes in the electric potential across the retina during light stimulation. When light stimulates the retina, a series of chemical reactions lead to changes in the electric charge across the retinal tissue, which can be measured non-invasively using ocular surface electrodes^1^. However, recording the ERG remotely at the cornea often yields small signals that are susceptible to interferences and noises. Specific setups and equipment must be used in clinical applications to obtain clean and reproducible results, which have been standardised in guidelines published by The International Society for Clinical Electrophysiology of Vision (ISCEV)^2^.

A common challenge encountered during clinical ERG recordings is the presence of electromagnetic noise, typically coming from the power supply network^3–6^. These electromagnetic fields can interact with the electrode cables and the recording equipment, thereby distorting the ERG waveforms. Line noise is challenging to remove through post-hoc filtering since the 50/60Hz power supply frequencies overlap with the bandwidth of the recorded signals^5^. Additionally, offset potential drift is a commonly observed phenomenon during recording sessions that can alter the amplitude and peak times of the ERG signals^7^.

Several strategies have been proposed to improve the quality of clinical ERG signals by reducing the impact of powerline interference. These include the use of passive shielding, active shielding, and signal pre-amplification^6,8,9^. The notable noise reduction obtained through signal pre-amplification highlights the benefits of on-source signal amplification^6^. We hypothesize that further noise resistance could potentially be obtained by digitising the amplified signal and wirelessly transmitting the signal at source, thereby removing transmission lines and further electrically isolating the patient.

In this study, a novel electroretinogram recording system was developed and validated to demonstrate the utility of such on-source signal processing. We developed a device that amplifies the signal at source and digitises the signal for wireless transmission to the recording apparatus through Bluetooth Low Energy (BLE). Smartphones were used to receive the wireless signals and an app was devised for controlling the amplifier setup as well as displaying the recorded ERG signals. The performance of our device was compared to the same ERG recordings obtained by using a standard commercially available recording setup.

## Materials and methods

### System overview

**Fig. 1:**
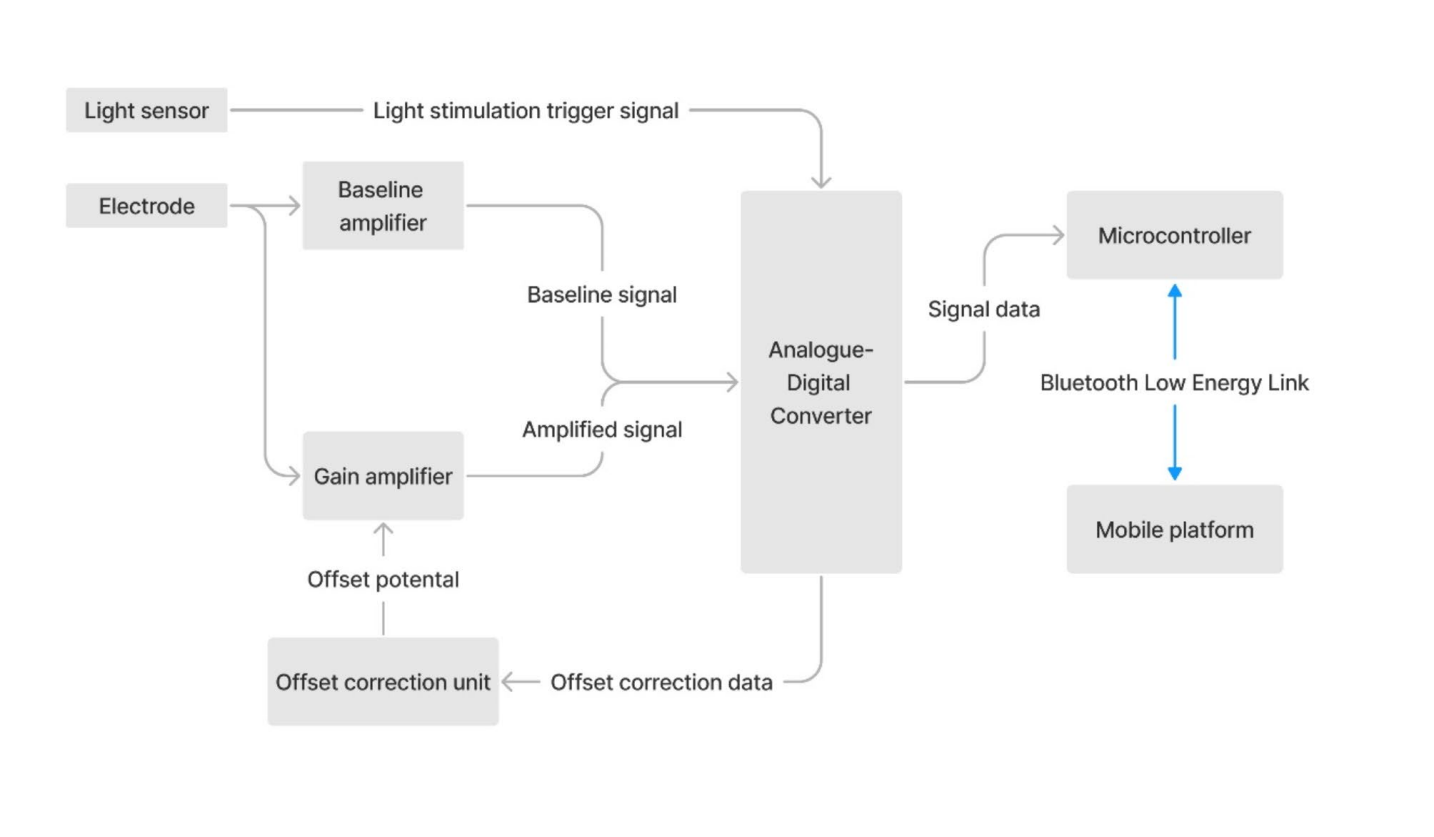
Structural overview of the novel recording system. The structural overview and function of the novel wireless ERG recording module. The bio-potential from the electrodes is first converted into digital data with offset correction. Digitalised data are packed and transmitted via Bluetooth low-energy and received by a smartphone. The smartphone reorganises the data packets to display the recorded ERG signals.

An overview of the novel wireless recording system is illustrated in Fig. 1. Similar to the traditional setup, two signal electrode leads and a ground electrode lead were required to acquire the differential signals. A light sensing module was used to synchronise the stimulation flash with the recorded signal. Signals from the electrode leads and the module were rerouted and multiplexed into three channels of analogue data, namely, the baseline signal, amplified signal, and light stimulation signals. Analogue signals were first amplified with a gain of 100 using an instrumentational amplifier (INA821, Texas Instrument, Dallas, TX) and thereafter converted into digital data with a 24 bits analogue-digital converter (ADS131M06, Texas Instrument, Dallas, TX) with simultaneous sampling of 32k sample per second per channel.

For offset correction, the digitised offset potential of the baseline signal was passed into a 16 bit digital-analogue converter (DAC80502, Texas Instrument, Dallas, TX) to create a reference level for the instrumentational amplifier of the amplified signal. Potential offset of the amplified signal would thus be compensated to allow larger dynamic range and prevent saturation of the recording amplifier. The design also allowed for larger gain to be used thereby enhancing the signal-to-noise ratio and signal resolution. The use of analogue filtering circuits was deliberately avoided to preserve the signal waveform integrity.

The signal digitalisation and offset correction operations were managed by a microcontroller (CC2642R, Texas Instrument, Dallas, TX). A bespoke Real-Time Operating System (RTOS) firmware was developed for the microcontroller. The thread management functionality of the firmware could effectively handle the signal processing, temporary data storage, and wireless communication functions. Bluetooth Low Energy (BLE) was selected as the wireless transmission protocol for the device since this protocol is commonly supported in most mobile devices with low computational power requirements.

An iOS application was developed for acquiring and displaying the recorded signals, controlling recording tasks and device configuration. Upon task initiation, the microcontroller handled the signal-processing procedure and wrapped the recorded data into wireless communication packets. The packed data was then streamed and reconstructed in the mobile application. The electroretinogram signals were stored on the device for post-hoc data processing and visualisation.

### Animal experimental setup

To evaluate the novel ERG recording system, five healthy wild-type mice (C57BL/6J) were used. The experimental protocol was approved by the Animal Experimentation Ethics Committee of the Chinese University of Hong Kong. All experiments were performed in accordance with regulations. The study design is in accordance with the ARRIVE guidelines.

Animals were provided by the Laboratory Animal Service Center of the Chinese University of Hong Kong. Mice were housed with temperature and relative humidity kept at 19±3 °C and 55±10% respectively. A dark light cycle of 12 h:12 h was provided with a clean standard diet and water.

Mice were first placed overnight in dark adaptation. Following all scotopic stimulations, the animals were light adapted for 10 min. The mice were anaesthetised at the beginning of the ERG recording session. Anaesthesia was performed with intraperitoneal injection of Ketamine-Xylaxine mixture with dosage of 100 mg/kg of Ketamine and 10 mg/kg of Xylazine. Gold wires were placed on the cornea with a reference electrode placed in the mouth. Gel was applied on the corneal surface for better conduction and electrode adhesion. A thin subcutaneous silver needle was used for the ground electrode.

### Electroretinogram evaluation setup

The signal recording performance of the novel wireless system was compared with the Espion E3 (Diagnosys LLC., Lowell, MA) recording system. Identical light stimulation and recording configurations were applied to both setups. For the dark-adapted setup, stimulation intensities 0.001 Cd.s.m^−2^, 0.1 Cd.s.m^−2^, and 10 Cd.s.m^−2^ were used. For the light-adapted setup, stimulation intensities of 1 Cd.s.m^−2^ were used. An additional flicker setup was performed in the light-adapted state with an intensity of 1 Cd.s.m^−2^ and a frequency of 10 Hz. The amplitudes and peak times were identified from the corresponding a-wave and b-wave from the ERG signals and compared between the two systems. Additional analyses were performed to evaluate the offset potential drift and noise power of ERG signals. Specific algorithms were designed for the calculation and comparison of the offset potential drift and power line noise power spectral density of the ERG signals.

### Data processing

An identical data processing flow was applied to both systems. Five signals were recorded at each light intensity and all signals were exported into MATLAB (MATLAB 2023a, MathWorks, Natick, MA) for further post-hoc processing and statistical analysis. Averaging and filtering were not implemented in the signal-acquisition flow.

Estimation of ERG signal properties among animal subjects, including amplitudes and peak times of the a-wave and b-wave, were extracted from the five original unprocessed signals. A MATLAB algorithm was applied to extract the amplitudes and peak time measurements from the a-wave and b-wave components. The median values were then used for statistical analysis.

The offset potential drift was estimated using linear model fitting of the signal. The offset potential drift of the signal is defined as the change in potential across the signal recording timespan, regardless of electrophysiological events. This relative potential change from light stimulation time zero can be described by a linear function without a y-intercept. The offset potential drift in the recorded ERG signals, in terms of the slope of the linear function, was estimated using a MATLAB linear model fitting algorithm with the least mean square error criterion. A larger slope implies a prominent offset potential drift in the recorded ERG signals. The magnitudes of the slope values were then used for statistical comparisons between the novel and reference systems.

Estimation of the powerline noise was performed by power spectral density estimation on the power supply frequency. Line noise components can be visualised as 50 Hz sinusoidal noise superimposed onto the ERG signals. The estimation of the line noise component was performed using a MATLAB Discrete Fast Fourier Transform (nFFT) algorithm followed by the extraction of the power spectral density of the 50 Hz component. A higher power spectral density indicates prominent powerline noise in the recorded ERG signals. The power spectral densities were used for statistical comparisons between the novel and reference systems.

### Data analysis

The signal properties for the evaluation of the novel system, including the signal amplitudes, signal peak times, offset potential drift, and powerline noise performance, were first evaluated using descriptive statistics. Confounding factors were identified using correlation analysis. The identified confounding factors were adjusted for in statistical analyses, which were performed using SPSS software. (SPSS 29.0.0, IBM, Armond, NY).

For the comparison of ERG signal properties in animal subjects, the median values of the signal properties were used for statistical analysis to account for the variation in the subject influence. For the comparison of offset potential drift and powerline noise, measurements from all results were used, as the variation of these two properties was from the study system. Parametric tests were used for comparisons if the results were normally distributed. Equal variances were not assumed. A two-tailed significance level of 0.05 was used.

## Results

### Descriptive analysis

The normality of the signal properties was evaluated using a normality test. Homogenous normality was not reported in the signal properties; hence, less robust non-parametric tests were used in the statistical analysis. No confounding factors for signal properties were identified.

### ERG signal waveform

**Fig. 2:**
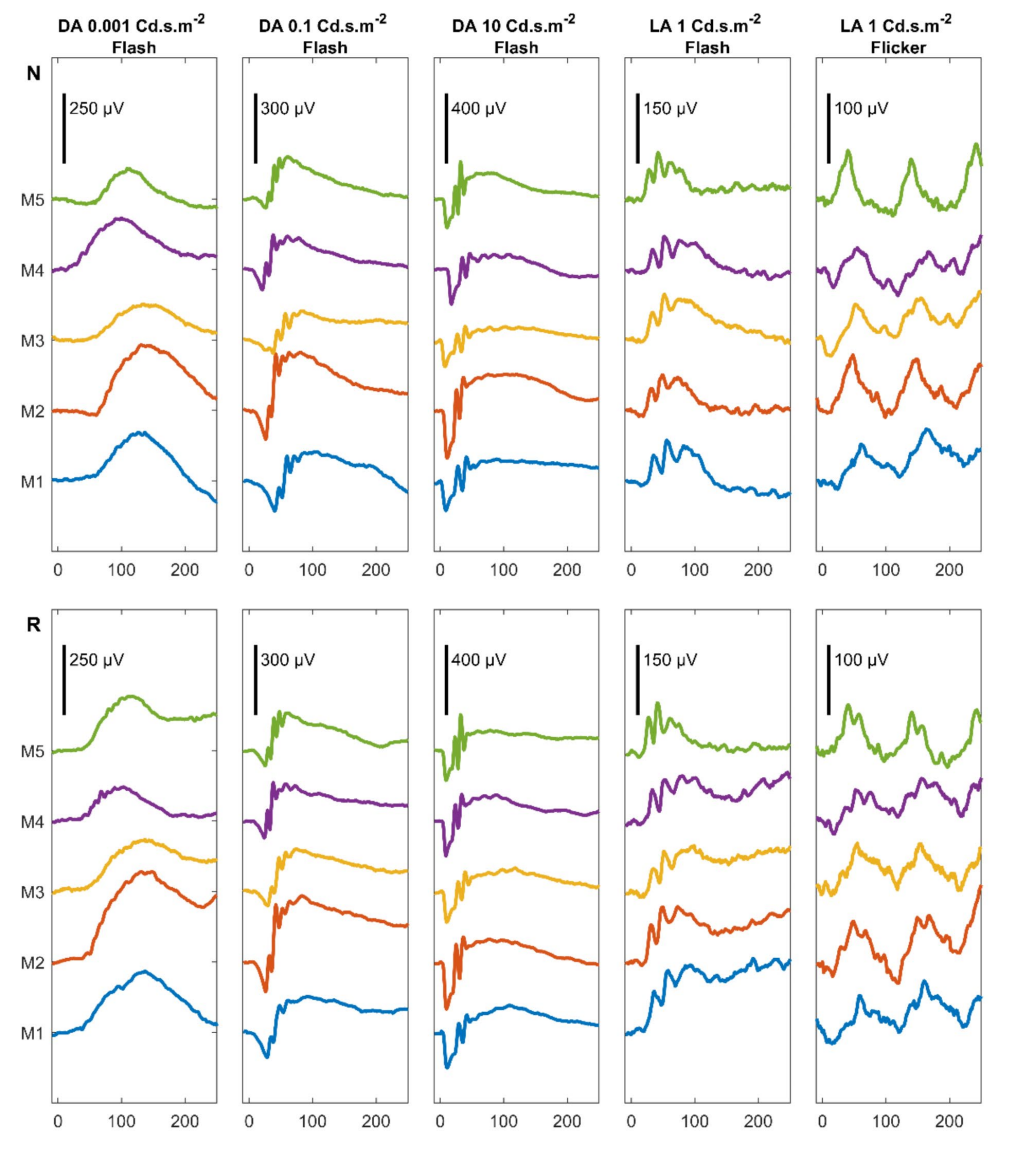
Electroretinogram data set from animal experiment. ERG recordings from five animals (M1-M5) are shown for each stimulation strength in dark adapted (DA) and light adapted (LA) conditions. The records obtained with the novel recording system are displayed at the top (N), and the reference recording system is displayed below (R). Signals with pre-stimulus time of 10ms and post-stimulus time of 250ms were displayed.

The ERG signals from the animal subjects recorded by the novel and reference systems are illustrated in Fig. 2. The ERG signals recorded by the novel system were robust and reproducible with easily identifiable a- and b-waves. Signal waveforms from the novel system were found to be highly coherent with the corresponding signals from the reference system among the study subjects.

Despite the analogous ERG signal waveform, two features were identified in the signals recorded by the novel system. First, changes in the offset potential were less prominent. Second, the small wavelet noise was less intensive, particularly under a light-adapted flicker setup. These two features were further analysed in the evaluation of offset potential drift and powerline noise.

### Comparison of signal properties

**Fig. 3:**
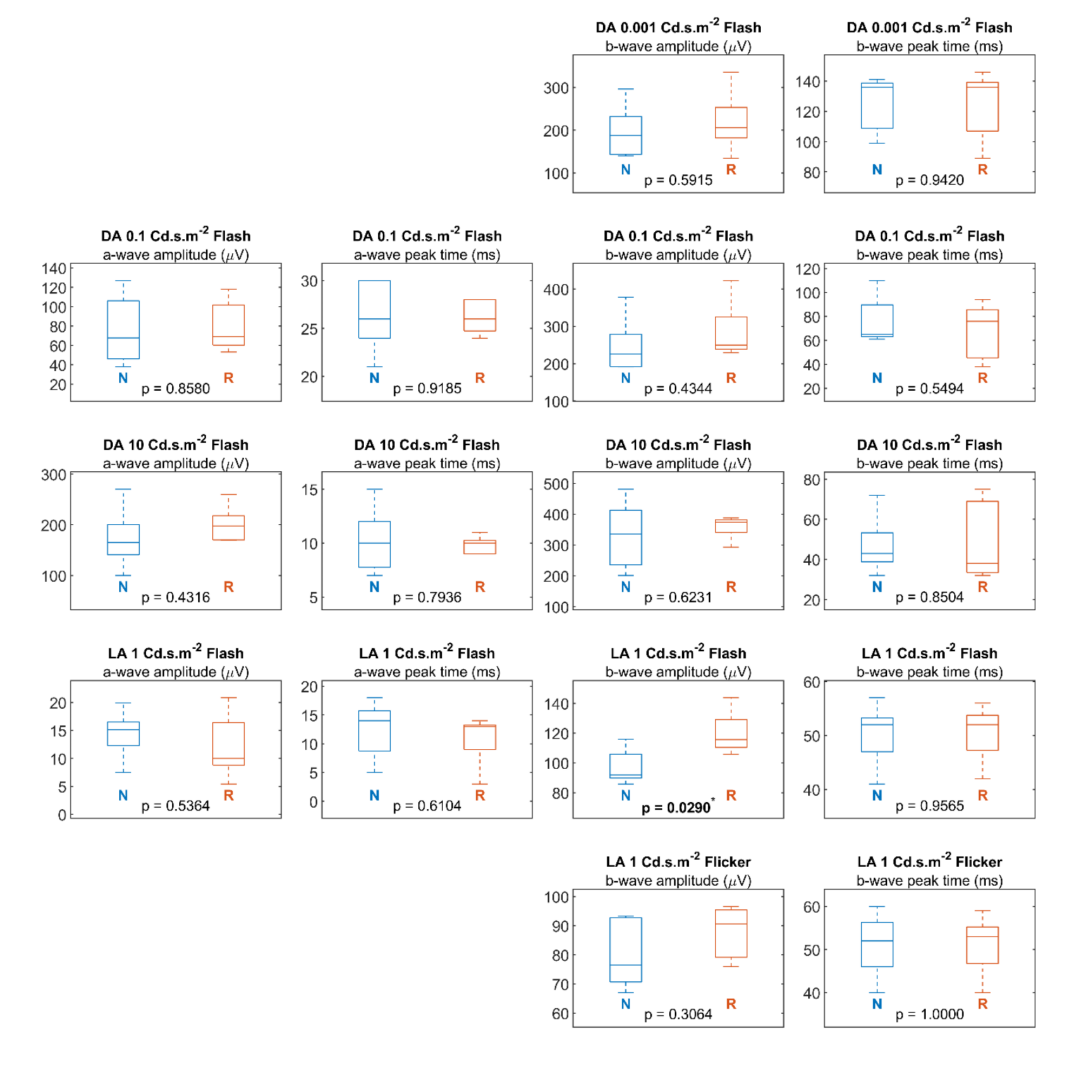
Distribution of Electroretinogram signal properties. Distribution of the subject ERG signal properties, including the amplitudes and peak times of a-wave and b-wave, were illustrated in the boxplot. N indicates the ERG signal properties recorded by the novel system. R indicates the ERG signal properties recorded by the reference system.

The distributions of the subject medians for ERG signal properties between the novel and reference systems are illustrated in Fig. 3. Comparisons were arranged by signal properties in columns and stimulation setup in rows. The N and R markers indicate the signal properties of the novel and reference systems, respectively. The Kruskal-Wallis test was used for the distribution analyses. The *P*-values for the analyses were shown in the boxplots.

Consistent with the observations of ERG signals among the subjects in Fig. 2, comparisons of signal properties were expected to be insignificant, as the signals from both systems were highly analogous. The distribution of signal properties from both systems overlapped without statistical significance in the a-wave amplitude (*P* > 0.43), a-wave peak time (*P* > 0.61), and b-wave peak time (*P* > 0.55). A significant difference was solely reported in the b-wave amplitude in the light adapted 1 Cd.s.m-2 setup (novel system 97.44µV ± 11.98; reference system 120.22 ± 14.76µV; *P* = 0.03). The significant difference in b-wave amplitudes can be explained by the prominent offset potential drift in the signals recorded by the reference system. The influence of the offset potential drift on the signal properties was quantitatively evaluated by analysing the offset potential drift.

### Analysis of offset potential drift

**Fig. 4:**
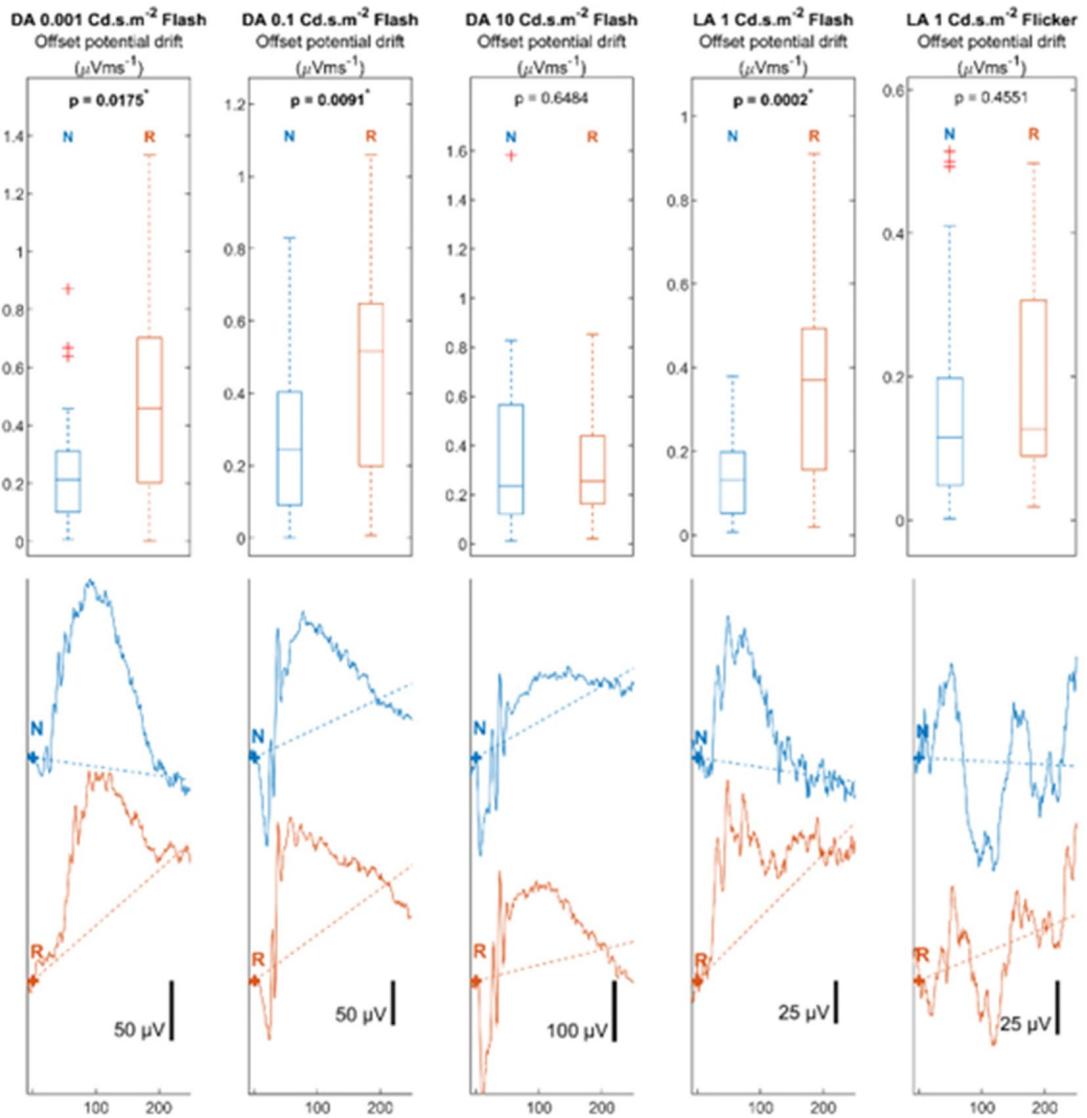
Comparison of offset potential drift in Electroretinogram signal. The slope of the fitted linear model for the offset potential drift estimation from the two systems is shown in the boxplot in the first row. The ERG signals corresponding to the mean slope value in the boxplot were illustrated to visualise the influence of the offset potential drift on the ERG signals in the second row. The fitted linear model was indicated by the dotted line. Signals with pre-stimulus time of 10ms and post-stimulus time of 250ms were displayed. N indicates the estimated potential drift and signal recorded by the novel system. R indicates the estimated potential drift and signal recorded by the reference system.

Using the linear model fitting method, the offset potential drifts of the original signals were extracted as the absolute values of the linear function slope. The distribution of the estimated values between the two systems were illustrated in Fig. 4. The distributions of the offset potential drift values were illustrated in the box plots in the first row. The ERG signals corresponding to the mean slope value from the box plots were plotted with the fitted linear model in the second row to visualise the offset potential drift influence on the ERG signals. Plots were arranged by stimulation setups in columns. The N and R markers indicate the signal properties of the novel and reference systems, respectively. The Kruskal-Wallis test was used for the distribution analyses. The *P*-values for the analyses are shown in the boxplot.

Consistent with the observations of ERG signals among subjects in Fig. 2, offset potential drift was expected to be less in signals recorded by the novel system. Distribution of the offset potential drift in novel system signal were significantly smaller in dark adapted 0.001 Cd.s.m^−2^ setup (novel system 0.26 ± 0.21 µVms^−1^; reference system 0.48 ± 0.34 µVms^−1^; *P* = 0.01), dark adapted 0.1 Cd.s.m^−2^ setup (novel system 0.25 ± 0.21 µVms^−1^; reference system 0.46 ± 0.29 µVms^−1^; *P* = 0.01) and light adapted 1 Cd.s.m^−2^ setup (novel system 0.13 ± 0.10 µVms^−1^; reference system 0.38 ± 0.27 µVms^−1^; *P* = 0.01). The differences in offset potential drift between the systems were significant and robust, which may bias the measurement of the ERG signal properties.

Taking signals from the light-adapted 1 Cd.s.m^−2^ setup as an example, given that the mean b-wave peak time is 52ms, the difference in the offset potential drift would lead to a difference in the b-wave amplitude of 13 µV. The significant observation of the b-wave amplitude illustrated in Fig. 3 may be rendered invalid when the amplitude bias from the offset potential drift was considered.

### Analysis of powerline noise

**Fig. 5:**
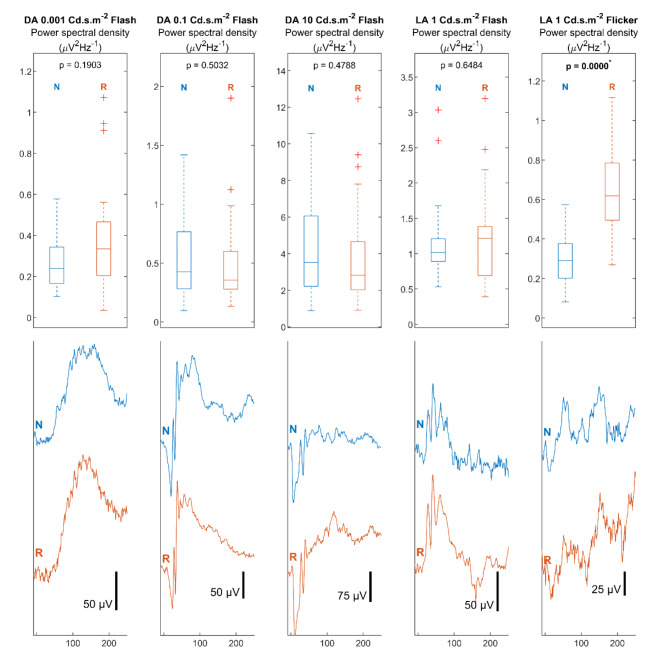
Comparison of line noise (50 Hz) power spectral density in Electroretinogram signal. The power spectral density of the 50 Hz noise component in the ERG signal from the two systems were illustrated in the boxplot in the first row. The ERG signals corresponding to the mean power spectral density in the boxplot are illustrated to visualise the influence of 50 Hz line noise power on the ERG signals in the second row. Signals with pre-stimulus time of 10ms and post-stimulus time of 250ms were displayed. N indicates the estimated potential drift and signal recorded by the novel system. R indicates the estimated potential drift and signal recorded by the reference system.

The powerline noise was estimated as the magnitude of the 50 Hz component of the power spectral density in the ERG signals. The distributions of the estimated power line noise values for both systems were illustrated in Fig. 5. The distributions of the noise power spectral densities were illustrated in the box plots in the first row. The signals corresponding to the mean noise estimation from the box plots were plotted in the second row to visualise the influence of powerline noise on the signals. Plots were arranged by stimulation setups in columns. The N and R markers indicate the signal properties of the novel and reference systems, respectively. The Kruskal-Wallis test was used for the distribution analyses. The *P*-values for the analyses are shown in the boxplot.

In the experimental setup, potential sources of power-line interference were removed from the site. Therefore, the power spectral densities of the powerline noise between both systems were small and insignificant (*P* > 0.19). Significant difference was reported in light adapted flicker setup (novel system 0.30 ± 0.14 uV^2^Hz^−1^; reference system 0.64 ± 0.24 uV^2^Hz^−1^; *P* = 0.00). The difference in the powerline noise power can be visualised in the corresponding signal plot, where the signal from the reference system was more intensively contaminated with low-amplitude noise.

**Fig. 6:**
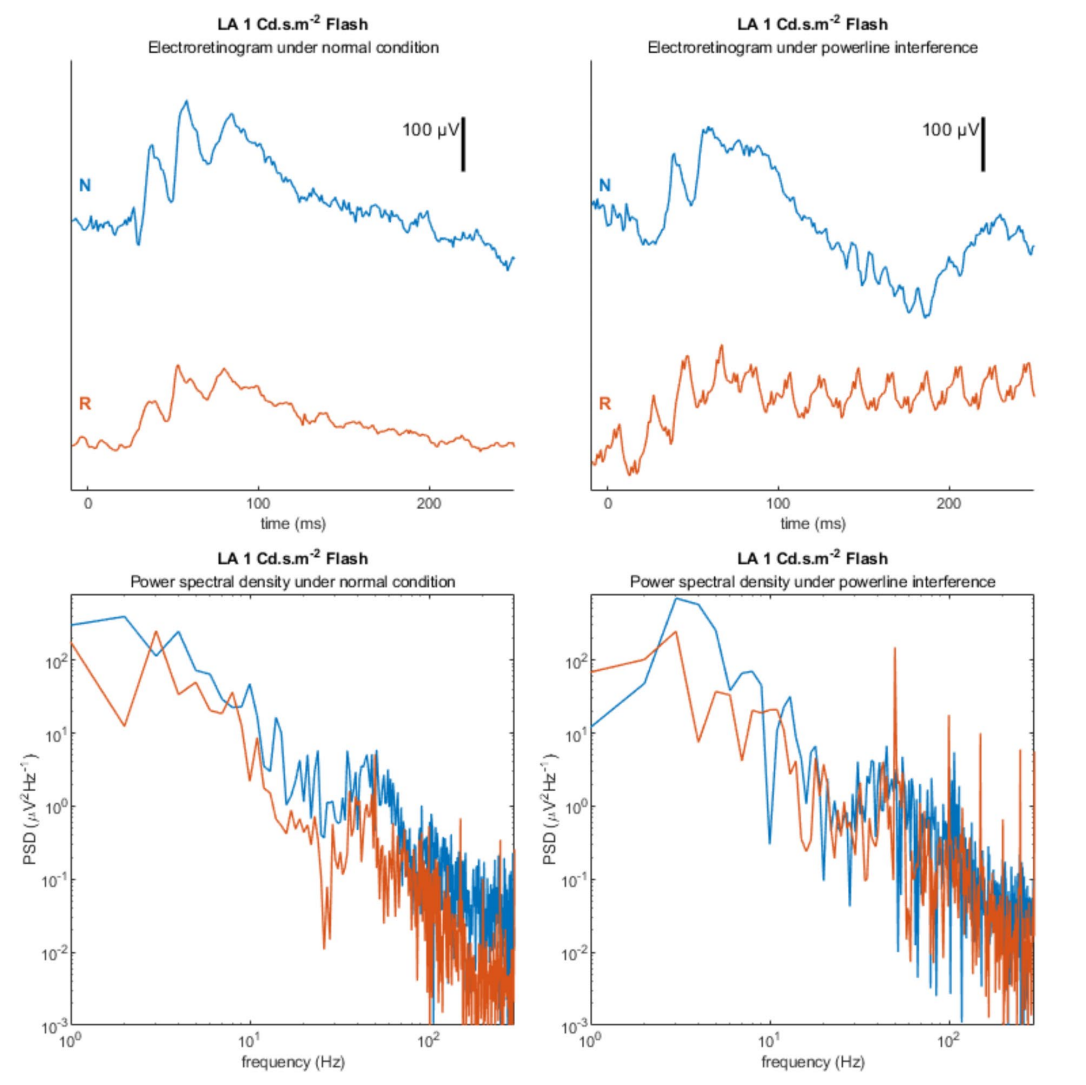
Comparison of ERG signal under normal condition and powerline interference. The ERG signals from the two systems are illustrated under normal conditions and prominent power line interference in the first row. The corresponding periodograms of the ERG signals were illustrated in the second row. Signals were obtained in subject M4 under a light-adapted 1 Cd.s.m^−2^ setup. N indicates the estimated potential drift and signal recorded by the novel system in the blue marker. R indicates the estimated potential drift and signal recorded by the reference system in the orange marker.

To validate the powerline noise resistance performance of the novel system from the on-source signal processing design, an additional signal recording session was performed on subject M4. Powerline interference was introduced by placing the computer power supply cable of the reference system close to the recording electrode. The recorded signals and corresponding power spectral densities are shown in Fig. 6. Signals from both systems were similar, with overlapping power spectral densities under normal conditions. However, with the introduction of powerline noise, the signals recorded by the reference system were heavily contaminated with periodic powerline noise. The spectral components of the noise and harmonics were clearly observed as peaks in the spectral plot. Under the same conditions, the signals recorded by the novel system were less affected by powerline noise. Such finding was coherent to our previous finding, which demonstrated the feasibility in utilising on-source signal processing design to reduce powerline noise in ERG signal^4^.

## Discussion

This report describes the development and validation of a novel wireless ERG recording module. The novel system recorded ERG signals with similar performance to a commercially available ERG equipment. The waveshapes were similar in both recording setups; however, the noise and drift in the wireless recording module were substantially less.

Our system measures only 50 × 30 mm and weighs less than 300 g, which is substantially smaller, lighter, and more portable than the commercially available ERG recording equipment. This miniaturisation is achieved through the use of a microcontroller with an integrated Bluetooth Low Energy protocol that provides wireless functionality for system control and data transmission. This wireless function makes the setup more convenient and less cumbersome for both technicians and patients.

For the novel system to be considered clinically useful and viable, the ERG signals need to be of comparable quality to those of clinical ERG systems. The signals recorded with the wireless system were similar to those from the commercially available recording system (see Figs. 2 and 3), with no statistically significant differences in signal amplitude or peak times. The diagnostic power of the two systems is hence considered to be comparable.

The important difference observed when using the wireless system was a marked reduction in offset potential drift. This was achieved by configuring the input amplifiers with proper input bias current compensation. As shown in Fig. 4, the offset potential drifts were less prominent in the signals recorded by the wireless system and was statistically significantly less than in the reference recording system (*P* < 0.02).

Our previous study showed the benefits in noise reduction when pre-amplifying ERG signals at source^6^ By also digitising and wirelessly transmitting the signal using on-source signal processing, further noise reductions can be achieved. The electrode transmission lines are now avoided which further reduces powerline noise interference. As shown in Fig. 5, the powerline noise in the wireless system was comparable to that in the reference system signal under low noise conditions. However, when there is significant noise interference, the enhanced noise-resistance of the wireless system resulted in statistically significant reduction in powerline noise interference as compared to the reference recording setup (*P* < 0.01). The combined improvement of offset potential drift and powerline noise enhances the performance and precision of the ERG signal recordings which improves the reliability of the clinical interpretation.

**Fig. 7:**
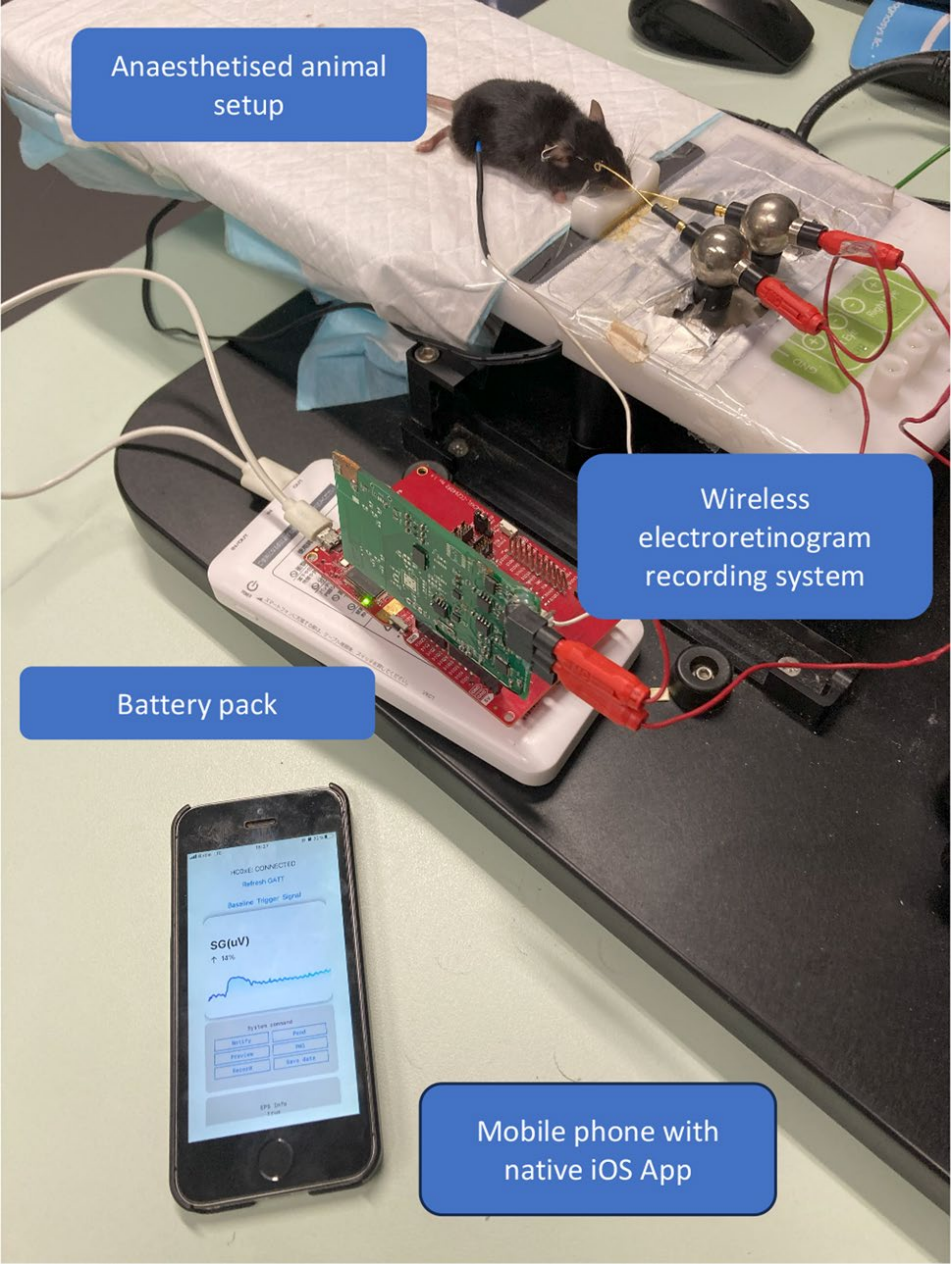
Illustration of the animal experiment setup. Gold wires were placed on the cornea with a reference electrode placed in the mouth of anaesthetised mouse and subcutaneous silver needle was used for the ground electrode. Signals recorded up by the novel system were transmitted wirelessly to a mobile phone running a bespoke native iOS app. Waveforms were visualised on the phone which also allowed the recording system to be controlled. The system is powered by a battery pack.

One limitation of our study is that the data were derived exclusively from animal experiments, shown in Fig. 7, and to date, no recordings have been conducted using our system in human subjects. The anaesthetised mouse model has been extensively used as the in-vivo animal setup for electrophysiology studies^10^. The model allows optimally controlled experiment conditions for consistent and reproducible electroretinogram results which are crucial for the preliminary performance evaluation of our novel device. The use of anesthetized animals in electroretinography (ERG) experiments offers advantages such as minimized movement artifacts, enhanced data quality, and a controlled environment, which facilitate reliable recordings. However, it also presents challenges, including altered physiological responses due to anaesthetic agents and different species responses^11^. Nevertheless, we believe that the results will be translational and applicable to human subjects as supported by previous studies demonstrating the consistency of waveform structures, functional correlations with histological data, and parallel aging effects across species, including human subjects^12,13^. These findings support the results obtained from animal electroretinography can be effectively translated to human contexts in the future.

This novel ERG recording setup has the potential to improve clinical ERG recordings in the future. As well as potentially improving the quality of ERG signals, the portable nature of the wireless device together with the smartphone app makes the system easy to implement and convenient to use. Patients undergoing ERG testing will have greater freedom of movement and comfort as they will not be tethered by electrode cables. Our simplified design of the recording system also reduces the fabrication and maintenance costs of the hardware which makes the platform more cost effective.

## Conclusion

In this study, a novel wireless electroretinogram recording system is developed and validated. Our results show that signals recorded with the wireless system are comparable to those measured with a commercially available recording system with no statistically significant difference in amplitudes and peak times of the recorded signals. However, the combination of on-source amplification and wireless transmission reduces noise interference and improves the quality of recorded signals. In addition, using on-source signal processing, we could reduce potential drift, thus further enhancing the quality of recorded signals. Our results demonstrate the feasibility of using simple hardware for measuring low-amplitude ERG signals and wirelessly transmitting this data to a smartphone. This study shows a proof of concept for enhancing the quality of ERG signals, and future studies are planned to validate this system on clinical recordings in patients.

## Data Availability

The datasets used and analysed during the current study available from the corresponding author on reasonable request.

## References

[1] Perlman, I. In *Webvision: The Organization of the Retina and Visual System* (eds Kolb, H., Fernandez, E. & Nelson, R.) (University of Utah Health Sciences Center, 1995). 21413389

[2] Robson, A. G. et al. ISCEV Standard for full-field clinical electroretinography (2022 update). *Doc. Ophthalmol.* **144**, 165–177. 10.1007/s10633-022-09872-0 (2022). 35511377 10.1007/s10633-022-09872-0 PMC9192408

[3] Gauvin, M., Lina, J. M. & Lachapelle, P. Advance in ERG analysis: From peak time and amplitude to frequency, power, and energy. *Biomed. Res. Int.* **2014**, 246096. 10.1155/2014/246096 (2014). 10.1155/2014/246096 PMC4100345 25061605

[4] Komáromy, A. M. et al. Technical issues in electrodiagnostic recording. *Vet. Ophthalmol.* **5**, 85–91. 10.1046/j.1463-5224.2002.00229.x (2002). 12071864 10.1046/j.1463-5224.2002.00229.x

[5] Ledolter, A. A., Todorova, M. G., Schoetzau, A. & Palmowski-Wolfe, A. M. Impact of a digital power line filter in the 2-global-flash multifocal electroretinogram of glaucoma patients compared to controls. *Curr. Eye Res.* **41**, 70–78. 10.3109/02713683.2014.1002043 (2016). 25612055 10.3109/02713683.2014.1002043

[6] Yip, Y. W. Y., Man, T. C., Pang, C. P. & Brelén, M. E. Improving the quality of electroretinogram recordings using active electrodes. *Exp. Eye Res.* **176**, 46–52. 10.1016/j.exer.2018.06.007 (2018). 29908144 10.1016/j.exer.2018.06.007

[7] Tang, J., Hui, F., Coote, M., Crowston, J. G. & Hadoux, X. Baseline detrending for the photopic negative response. *Transl. Vis. Sci. Technol.* **7**, 9. 10.1167/tvst.7.5.9 (2018). 30258702 10.1167/tvst.7.5.9 PMC6152608

[8] Jiang, Y. et al. Effective biopotential signal acquisition: Comparison of different shielded drive technologies. *Appl. Sci.* **8**, 276 (2018).

[9] van Metting, A. C., Peper, A. & Grimbergen, C. A. High-quality recording of bioelectric events. Part 1. Interference reduction, theory and practice. *Med. Biol. Eng. Comput.* **28**, 389–397. 10.1007/bf02441961 (1990). 2277538 10.1007/BF02441961

[10] Liu, P. K., Huang, W. C. & Wang, N. K. Electroretinogram (ERG) to evaluate the retina using mouse models. *Methods Mol. Biol.* **2560**, 217–227. 10.1007/978-1-0716-2651-1_20 (2023). 36481898 10.1007/978-1-0716-2651-1_20 PMC12409681

[11] Charng, J. et al. Conscious wireless electroretinogram and visual evoked potentials in rats. *PLoS One* **8**, e74172. 10.1371/journal.pone.0074172 (2013). 24069276 10.1371/journal.pone.0074172 PMC3771909

[12] Chang, B. Mouse models for studies of retinal degeneration and diseases. *Methods Mol. Biol.* **935**, 27–39. 10.1007/978-1-62703-080-9_2 (2013). 23150358 10.1007/978-1-62703-080-9_2 PMC3856760

[13] Heckenlively, J. R., Winston, J. V. & Roderick, T. H. Screening for mouse retinal degenerations. I. Correlation of indirect ophthalmoscopy, electroretinograms, and histology. *Doc. Ophthalmol.* **71**, 229–239. 10.1007/bf00170972 (1989). 2776628 10.1007/BF00170972

